# Cross-sectional associations of lipid accumulation and immune–nutritional indices with ultrasonography-detected gallstone disease: an explainable machine learning analysis

**DOI:** 10.3389/fnut.2026.1879120

**Published:** 2026-09-09

**Authors:** Lei Wang, Hao Sun, Cai Wang, Yuantao Qi, Xue Gong

**Affiliations:** 1 Chongqing University Three Gorges Hospital, Chongqing, China; 2 Qingdao Municipal Hospital, University of Health and Rehabilitation Sciences, Qingdao, China; 3 Binhai County People's Hospital, Yancheng, Jiangsu, China; 4 Shandong Cancer Hospital and Institute, Shandong First Medical University and Shandong Academy of Medical Sciences, Jinan, China

**Keywords:** CALLY index, cholelithiasis, gallstone disease, lipid accumulation product, machine learning, prognostic nutritional index, TyG-WC, waist-triglyceride index

## Abstract

**Background:**

Recent studies have linked individual lipid accumulation and triglyceride–glucose indices to the prevalence of gallstones, but direct comparisons of metabolic, inflammatory, and immune–nutritional indices in hospital-based health examination cohorts with ultrasonography-defined gallstones remain limited. We therefore examined whether routinely available composite indices were associated with prevalent gallstone disease and evaluated their cross-sectional discrimination performance.

**Methods:**

We conducted a single-center, hospital-based cross-sectional study of adults undergoing routine health examinations at the Health Examination Center of the Second Affiliated Hospital of Shandong First Medical University during 2024–2025. The primary inferential analyses included multivariable logistic regression models evaluating the associations between each candidate index and ultrasonography-detected gallstone disease. Quartile analyses, restricted cubic splines, ROC analyses, calibration, decision curve analysis, and explainable machine learning analyses were treated as exploratory. Stratified 5-fold cross-validation was used to assess internal model stability.

**Results:**

Among 1,376 participants [mean (SD) age, 47.27 (13.35) years; 743 men (54.00%)], 127 (9.23%) had ultrasonography-detected gallstone disease. In Model 3, TyG-WC was nominally associated with gallstone disease per 1-SD increment (OR, 1.33; 95% CI, 1.05–1.69; *p* = 0.017) and in Q4 versus Q1 (OR, 3.10; 95% CI, 1.38–6.96; *p* = 0.006), although the quartile-specific CIs were wide. The CALLY index (OR per 1-SD, 0.62; 95% CI, 0.46–0.84; *p* = 0.002), NLR (OR, 1.30; 95% CI, 1.09–1.55; *p* = 0.004), and PNI (OR, 0.74; 95% CI, 0.61–0.91; *p* = 0.004) remained significant after within-family false discovery rate (FDR) correction, whereas TyG-WC and CTI did not. TyG-WC had limited single-marker discrimination (AUC, 0.66; 95% CI, 0.61–0.71). Penalized logistic regression showed only modest internally validated discrimination (5-fold cross-validated AUC, 0.74; 95% CI, 0.69–0.78), with low precision–recall performance.

**Conclusion:**

Several routinely derived metabolic and immune–nutritional indices were associated with prevalent ultrasonography-detected gallstone disease, but effect estimates were imprecise for some comparisons and discrimination was modest. The findings are hypothesis-generating; they do not establish incremental predictive value beyond conventional risk factors or support stand-alone clinical risk stratification without external and longitudinal validation.

## 1 Introduction

Gallstone disease is one of the most prevalent hepatobiliary disorders worldwide and is frequently detected during routine abdominal ultrasonography in ostensibly asymptomatic adults (1–6). Although gallstones may remain clinically silent, they can lead to biliary colic, acute cholecystitis, choledocholithiasis, cholangitis, pancreatitis, and substantial healthcare utilization. Contemporary epidemiologic evidence indicates that the burden of gallstone disease is increasing in parallel with obesity, aging, dyslipidemia, insulin resistance, and metabolic dysfunction (1–5).

The pathogenesis of cholesterol gallstones involves hepatic cholesterol hypersecretion, bile supersaturation, impaired gallbladder motility, mucin hypersecretion, and nucleation of cholesterol crystals (6–9). Insulin resistance, central adiposity, triglyceride-rich lipoprotein metabolism, low-grade inflammatory signaling, and fatty liver may contribute to these processes (7–10). Accordingly, metabolic and inflammatory measurements obtained during routine health examinations may capture systemic correlates of gallstone disease, although cross-sectional associations cannot establish causal pathways.

Traditional risk assessment often relies on single measurements such as BMI, waist circumference, fasting glucose, triglycerides, or HDL-C. Composite indices may summarize related dimensions of central adiposity, lipid accumulation, insulin resistance, inflammation, and nutritional status. Several recent studies, particularly analyses of NHANES 2017–2020, have linked WTI, TyG-derived indices, LAP, and visceral lipid accumulation measures with gallstone prevalence using questionnaire-based gallstone histories (11–16). These data are informative but differ from a hospital cohort in which gallstones are ascertained concurrently by abdominal ultrasonography; we, therefore, avoid implying that all previous gallstone studies used self-report or lacked imaging.

The candidate metabolic indices were selected because they can be reconstructed from measurements routinely available in health examination practice and because they represent complementary, rather than identical, constructs. WTI combines waist circumference and triglycerides (11); TyG and its anthropometric derivatives combine fasting triglyceride–glucose burden with overall or central adiposity (12, 17–20); and LAP, VAI, CMI, and AIP reflect lipid accumulation, visceral adipose dysfunction, cardiometabolic burden, or atherogenic lipid balance (21–25). We used the original published formulas to preserve comparability with prior studies rather than recalibrating coefficients to the present cohort.

Inflammatory and immune–nutritional indices were selected based on the same routine-data principle. CTI combines hsCRP with TyG-related metabolic stress (26), whereas the CALLY index, NLR, PLR, MLR, SII, SIRI, and PNI summarize different aspects of systemic inflammation or albumin- and lymphocyte-based nutritional–inflammatory status (27–30). Their inclusion allowed a domain-level comparison of metabolic and immune–nutritional signals without requiring insulin assays, advanced body composition imaging, metabolomics, or specialized cytokine panels.

We therefore studied adults undergoing routine health examinations at the Health Examination Center of the Second Affiliated Hospital of Shandong First Medical University during 2024–2025. The primary outcome was prevalent ultrasonography-detected gallstone disease. The primary inferential objective was to estimate multivariable-adjusted associations between each routinely derived index and gallstone disease, with odds ratios and 95% CIs as the principal effect measures. We hypothesized that indices reflecting central lipid–glucose burden and impaired immune–nutritional balance would show associations after adjustment for demographic, lifestyle, and clinical background factors. Quartile analyses, spline visualization, single-marker ROC analysis, and machine learning analyses were considered exploratory.

The machine learning component was used mainly to examine internal discrimination and model-based feature contributions rather than to claim a deployable clinical prediction tool. SHAP analyses were therefore interpreted as explanations of model output, not as inferential tests or evidence of causality. This distinction is important because the cross-sectional design, relatively low event prevalence, and lack of external validation limit predictive claims.

## 2 Materials and methods

### 2.1 Study design and participants

This hospital-based, cross-sectional study used a consecutive convenience sample of adults who underwent a single routine health examination at the Health Examination Center of the Second Affiliated Hospital of Shandong First Medical University between 2024 and 2025. Among 8,346 initially screened attendees, 5,223 were excluded because gallbladder ultrasonography was unavailable; 254 because of acute hepatobiliary diseases, such as acute cholecystitis or cholangitis; 1,123 because key analysis variables were missing; 33 because of malignancy; six because of pregnancy; and 331 because of incomplete remaining covariates or implausible/extreme measurements identified during data quality control. Therefore, the final analytic cohort comprised 1,376 participants. Participants with prior cholecystectomy were excluded from gallstone outcome classification. The final analytic dataset contained one record per participant, with no missing values among the variables included in the reported analyses.

### 2.2 Outcome definition

The primary outcome was ultrasonography-detected gallstone disease. Abdominal ultrasonography was performed by sonographers at the health examination center, and outcome status was determined based on the formal ultrasound report. Only definite gallbladder stones were classified as gallstone disease (gallstone = 1). Biliary sludge, microlithiasis, isolated gallbladder wall thickening, and other non-specific gallbladder findings were not classified as gallstones. Participants with prior cholecystectomy were excluded. As outcome and candidate indices were measured during the same health examination, the study estimates cross-sectional associations and discrimination rather than incidence, prognosis, or causal effects.

### 2.3 Variable construction

BMI was calculated as weight in kilograms divided by height in meters squared, and WHtR was calculated as waist circumference divided by height using the same length units. The Chinese overweight/obesity indicator used BMI > =24 kg/m^2. TG and fasting plasma glucose were converted to mg/dL when required by published formulas. Current smoking was defined as having smoked more than 100 cigarettes during the lifetime and still smoking at the time of examination. Regular alcohol drinking was defined as alcohol consumption at least once per week.

WTI was calculated as ln[TG (mg/dL) x waist circumference (cm)/2] (11). TyG was calculated as ln[TG (mg/dL) x fasting plasma glucose (mg/dL)/2] (17, 18). TyG-BMI, TyG-WC, and TyG-WHtR were calculated as TyG multiplied by BMI, waist circumference, and WHtR, respectively (12, 19, 20). LAP was calculated as (waist circumference - 65) x TG for men and as (waist circumference - 58) x TG for women, with TG in mmol/L (21). We retained these originally published sex-specific LAP constants for comparability and did not recalibrate them for the present Chinese cohort. VAI was calculated as [WC/(39.68 + 1.88 x BMI)] x (TG/1.03) x (1.31/HDL-C) for men and as [WC/(36.58 + 1.89 x BMI)] x (TG/0.81) x (1.52/HDL-C) for women, with TG and HDL-C in mmol/L (22). CMI was calculated as TG/HDL-C x WHtR (23), while AIP was calculated as log10(TG/HDL-C) with both lipid concentrations expressed in mmol/L (24, 25). A complete formula summary is provided in Supplementary Table S1.

Remnant cholesterol (RC) was calculated as total cholesterol - LDL-C - HDL-C, and RCII was calculated as RC x hsCRP/10. CTI was calculated as TyG + 0.412 x ln(hsCRP) according to the published CRP-TyG framework (26). The CALLY index was calculated as albumin (g/L) x lymphocyte count (10^9/L) / hsCRP (mg/L) (27). NLR, PLR, and MLR were defined as the neutrophil-to-lymphocyte, platelet-to-lymphocyte, and monocyte-to-lymphocyte ratios, respectively. SII was calculated as platelet count x neutrophil count/lymphocyte count, SIRI as neutrophil count x monocyte count/lymphocyte count, and PNI as albumin (g/L) + 5 x lymphocyte count (10^9/L) (28–30).

### 2.4 Covariates

Covariates were chosen to characterize demographic, lifestyle, cardiometabolic, hepatobiliary, purine metabolic, and kidney function profiles. Model 1 included age and sex; Model 2 additionally included current smoking and regular alcohol drinking; and Model 3 further included systolic and diastolic blood pressure, ultrasonography-detected fatty liver, ALT, GGT, uric acid, and eGFR. The eGFR was retained as an extended comorbidity-background variable rather than being considered an established causal confounder of gallstones. A sensitivity analysis excluding the eGFR produced materially similar estimates for the principal associated indices. Components that mathematically define a composite index were not simultaneously entered with that index because of structural coupling and collinearity. This modeling choice improves numerical stability but does not permit separation of the composite association from the residual effects of its individual components, which we acknowledge as a limitation.

### 2.5 Statistical analysis

Continuous variables are presented as means (SD), and categorical variables as numbers (%). Between-group comparisons were performed using Welch’s *t*-test for continuous variables because this approach does not require equal group variances and is robust when group sizes differ, and the chi-squared test or Fisher’s exact tests were used for categorical variables, as appropriate. All tests were two-sided. Effect estimates and descriptive statistics were rounded to two decimal places, whereas *p*-values were reported to three decimal places and as *p* < 0.001 when less than 0.001. Statistical significance for nominal analyses was defined as a *p*-value of <0.05, with emphasis placed on effect sizes and 95% CIs rather than dichotomous significance alone.

Logistic regression was used to estimate ORs and 95% CIs for gallstone disease. Each index was analyzed separately as a standardized continuous variable and by quartiles, with Q1 as the reference. Model 1 was adjusted for age and sex; Model 2 was additionally adjusted for current smoking and regular alcohol drinking; and Model 3 was further adjusted for systolic and diastolic blood pressure, fatty liver, ALT, GGT, uric acid, and eGFR. P for trend was estimated using the quartile-specific median as a continuous term. As multiple related indices and secondary analyses were examined, the analyses were considered exploratory. As a multiplicity sensitivity analysis, the Benjamini–Hochberg false discovery rate (FDR) correction was applied to Model 3 *p*-values for continuous indices separately within the metabolic/lipid accumulation and inflammatory/immune–nutritional families. No *post hoc* power calculation was performed. The sample size was determined by the number of eligible participants available during the fixed study period rather than by an *a priori* effect size calculation.

Restricted cubic spline analyses were exploratory and were limited to six markers selected to represent the principal metabolic and immune–nutritional domains highlighted by the regression analyses and study rationale: TyG-WC, TyG-WHtR, WTI, LAP, CALLY index, and NLR. Curves used the Model 3 covariate structure. For visual stability, plots were displayed from the 2.5th to the 97.5th percentiles; truncation was applied only to the plotted range and not to model fitting. Both the overall association *p*-value and the non-linearity *p*-value are reported for each displayed spline. ROC analyses quantified single-marker discrimination and Youden index cutoffs, but these cutoffs were treated as descriptive and not as clinically validated thresholds.

Machine learning analyses were exploratory and were intended to assess internal discrimination and feature contribution rather than to produce a finalized clinical prediction tool. The original stratified 70:30 training–test split was retained as a secondary descriptive hold-out analysis; the ratio was chosen pragmatically to preserve a test sample while retaining most observations for training. As results from a single split can be unstable with 127 gallstone events, stratified 5-fold cross-validation was emphasized as the principal internal validation analysis. The predictor matrix included the routine clinical variables and derived indices used in the model interpretation analysis; participant identifiers and the outcome variable were excluded. Penalized logistic regression used standardized predictors, L2 regularization (C = 1.0), and balanced class weights. Random forest and extra trees models used class-weighted ensembles; gradient boosting used inverse-frequency sample weights. Hyperparameters were deliberately prespecified rather than extensively tuned: Tree ensembles used 200–500 estimators, square root feature sampling, and a minimum leaf size of 5, while gradient boosting used a learning rate of 0.05, shallow trees, and subsampling. The AUC and PR-AUC were emphasized because accuracy can be misleading with a 9.23% outcome prevalence. Calibration and decision curve results were interpreted descriptively. Random forest SHAp values were used only to explain model output; mean absolute SHAP values ranked contribution magnitude, while dependence plots were used to assess the direction of feature effects. Mean signed SHAP values and univariable *p*-values were not used to infer association direction or statistical significance.

## 3 Results

### 3.1 Participant characteristics

A total of 1,376 adults were included in the final complete-case analytic dataset after the exclusions described in Section 2.1. The mean (SD) age was 47.27 (13.35) years, and 743 participants (54.00%) were men. Ultrasonography-detected gallstone disease was present in 127 participants (9.23%). Participants with gallstones were substantially older than those without gallstones [57.02 (12.56) versus 46.28 (13.03) years; *p* < 0.001], underscoring age as an important source of confounding. They also had higher waist circumference, BMI, TyG-WC, TyG-WHtR, and NLR, as well as lower CALLY index and PNI values (all nominal *p* < 0.05; Table 1). The WHtR is used consistently to denote the waist-to-height ratio throughout the revised manuscript.

**Table 1:** Baseline characteristics according to ultrasonography-detected gallstone disease.

Characteristic	Gallstone disease			
	Overall	No	Yes	*p*-value
Overall	1,376	1,249	127	
Age, years	47.27 (13.35)	46.28 (13.03)	57.02 (12.56)	<0.001
Height, cm	164.80 (8.38)	164.68 (8.43)	165.92 (7.76)	0.092
Weight, kg	65.69 (10.28)	65.33 (10.23)	69.20 (10.20)	<0.001
Waist circumference, cm	84.33 (7.68)	83.97 (7.62)	87.94 (7.34)	<0.001
Systolic blood pressure, mmHg	118.50 (13.15)	118.09 (13.12)	122.46 (12.88)	<0.001
Diastolic blood pressure, mmHg	73.34 (8.50)	73.00 (8.41)	76.69 (8.66)	<0.001
Neutrophils, 10^9/L	3.46 (0.68)	3.45 (0.67)	3.55 (0.69)	0.114
Lymphocytes, 10^9/L	1.97 (0.39)	1.98 (0.39)	1.88 (0.40)	0.006
Monocytes, 10^9/L	0.40 (0.10)	0.40 (0.10)	0.38 (0.09)	0.027
Platelets, 10^9/L	222.69 (44.12)	223.23 (43.88)	217.40 (46.29)	0.176
hsCRP, mg/L	1.40 (0.95)	1.35 (0.90)	1.83 (1.29)	<0.001
Albumin, g/L	45.07 (2.32)	45.15 (2.29)	44.34 (2.45)	<0.001
ALT, U/L	28.22 (12.20)	27.93 (11.99)	31.04 (13.88)	0.016
AST, U/L	24.01 (7.49)	23.91 (7.43)	24.97 (8.09)	0.161
GGT, U/L	34.33 (19.58)	34.25 (19.30)	35.08 (22.17)	0.685
Fasting plasma glucose, mmol/L	5.22 (0.77)	5.19 (0.75)	5.58 (0.87)	<0.001
Total cholesterol, mmol/L	4.62 (0.70)	4.60 (0.69)	4.75 (0.71)	0.022
Triglycerides, mmol/L	1.60 (0.81)	1.58 (0.80)	1.80 (0.83)	0.005
HDL-C, mmol/L	1.32 (0.20)	1.32 (0.20)	1.28 (0.20)	0.018
LDL-C, mmol/L	2.71 (0.60)	2.70 (0.59)	2.86 (0.62)	0.006
Uric acid, umol/L	330.55 (71.53)	329.39 (71.60)	342.02 (70.16)	0.055
Creatinine, umol/L	69.46 (12.61)	69.20 (12.53)	72.02 (13.14)	0.022
eGFR, mL/min/1.73 m^2	101.60 (12.54)	102.42 (12.43)	93.57 (10.68)	<0.001
BMI, kg/m^2	24.10 (2.63)	24.01 (2.63)	25.04 (2.45)	<0.001
WHtR	0.51 (0.05)	0.51 (0.05)	0.53 (0.04)	<0.001
TyG index	8.68 (0.53)	8.66 (0.53)	8.88 (0.50)	<0.001
TyG-BMI	209.80 (30.47)	208.50 (30.41)	222.60 (28.13)	<0.001
TyG-WC	733.79 (94.63)	728.94 (93.86)	781.51 (89.10)	<0.001
TyG-WHtR	4.46 (0.56)	4.43 (0.56)	4.71 (0.52)	<0.001
WTI	8.58 (0.51)	8.56 (0.51)	8.75 (0.47)	<0.001
LAP	37.38 (25.36)	36.41 (24.86)	46.90 (28.22)	<0.001
VAI	1.92 (1.08)	1.89 (1.08)	2.20 (1.13)	0.003
CMI	0.66 (0.43)	0.65 (0.42)	0.78 (0.48)	0.003
AIP	0.04 (0.23)	0.03 (0.23)	0.11 (0.22)	<0.001
RC, mmol/L	0.58 (0.34)	0.58 (0.34)	0.62 (0.37)	0.300
RCII	0.09 (0.09)	0.08 (0.09)	0.12 (0.11)	0.001
CTI	8.74 (0.63)	8.71 (0.63)	9.05 (0.60)	<0.001
CALLY index	94.58 (70.11)	97.38 (71.38)	67.04 (48.32)	<0.001
NLR	1.80 (0.40)	1.78 (0.39)	1.94 (0.42)	<0.001
PLR	117.99 (34.93)	117.61 (34.63)	121.66 (37.67)	0.247
MLR	0.21 (0.05)	0.21 (0.05)	0.21 (0.05)	0.686
SII	399.97 (119.23)	397.69 (117.97)	422.41 (129.31)	0.040
SIRI	0.73 (0.25)	0.72 (0.25)	0.75 (0.25)	0.258
PNI	54.92 (3.16)	55.04 (3.12)	53.72 (3.30)	<0.001
Sex				0.230
Female	633 (46.00)	581 (46.52)	52 (40.94)	
Male	743 (54.00)	668 (53.48)	75 (59.06)	
Current smoking				0.773
No	1,065 (77.40)	968 (77.50)	97 (76.38)	
Yes	311 (22.60)	281 (22.50)	30 (23.62)	
Regular alcohol drinking				0.524
No	1,050 (76.31)	956 (76.54)	94 (74.02)	
Yes	326 (23.69)	293 (23.46)	33 (25.98)	
Fatty liver				0.008
No	904 (65.70)	834 (66.77)	70 (55.12)	
Yes	472 (34.30)	415 (33.23)	57 (44.88)	
Hypertension				0.037
No	1,268 (92.15)	1,157 (92.63)	111 (87.40)	
Yes	108 (7.85)	92 (7.37)	16 (12.60)	
Diabetes				<0.001
No	1,339 (97.31)	1,223 (97.92)	116 (91.34)	
Yes	37 (2.69)	26 (2.08)	11 (8.66)	
Dyslipidemia				<0.001
No	719 (52.25)	672 (53.80)	47 (37.01)	
Yes	657 (47.75)	577 (46.20)	80 (62.99)	
Chinese overweight				<0.001
No	669 (48.62)	628 (50.28)	41 (32.28)	
Yes	707 (51.38)	621 (49.72)	86 (67.72)	

WHtR denotes waist-to-height ratio. Current smoking was defined as >100 lifetime cigarettes with ongoing smoking; regular alcohol drinking was defined as > = 1 drinking occasion per week; and Chinese overweight/obesity was defined as BMI > = 24 kg/m^2. *p*-values are reported to three decimal places or as <0.001.

### 3.2 Multivariable associations of metabolic and lipid accumulation indices

The marked age difference between the outcome groups attenuated several crude metabolic associations. For example, the unadjusted OR per 1-SD increase in TyG-WC was 1.72 (95% CI, 1.43–2.06; *p* < 0.001), decreasing to 1.35 (95% CI, 1.08–1.68; *p* = 0.007) after age and sex adjustment and to 1.33 (95% CI, 1.05–1.69; *p* = 0.017) in Model 3. In Model 3, the TyG-WC quartile ORs were 2.41 (95% CI, 1.10–5.30; *p* = 0.029), 2.92 (95% CI, 1.34–6.36; *p* = 0.007), and 3.10 (95% CI, 1.38–6.96; *p* = 0.006) for Q2–Q4 versus Q1, respectively. These estimates had wide and substantially overlapping CIs; therefore, the trend *p* = 0.013 should not be interpreted as evidence of a precise graded dose–response relationship. The TyG-WHtR was borderline but not statistically significant as a continuous exposure in Model 3 (OR, 1.23; 95% CI, 0.99–1.53; *p* = 0.058). WTI was associated in the unadjusted analysis but was already non-significant after age and sex adjustment (OR, 1.16; *p* = 0.148) and remained non-significant in Model 3 (OR, 1.15; *p* = 0.205). LAP was non-significant as a continuous exposure in Model 3 (*p* = 0.208), whereas Q3 and Q4 were nominally associated; because P for trend was 0.114 and the spline did not provide strong evidence of non-linearity, the quartile finding was interpreted cautiously. Detailed estimates are shown in Table 2.

**Table 2:** Multivariable associations of metabolic and lipid accumulation indices with gallstone disease.

Variable	Model 1		Model 2		Model 3	
	OR (95% CI)	*p*-value	OR (95% CI)	*p*-value	OR (95% CI)	*p*-value
TyG						
Continuous	1.13 (0.92, 1.38)	0.262	1.13 (0.92, 1.39)	0.246	1.11 (0.89, 1.38)	0.351
Q1	Reference		Reference		Reference	
Q2	1.30 (0.67, 2.52)	0.444	1.29 (0.66, 2.52)	0.448	1.35 (0.69, 2.64)	0.383
Q3	1.80 (0.96, 3.35)	0.066	1.78 (0.96, 3.33)	0.068	1.67 (0.88, 3.15)	0.114
Q4	1.65 (0.88, 3.10)	0.119	1.66 (0.88, 3.12)	0.116	1.61 (0.84, 3.09)	0.150
P for trend		0.093		0.090		0.144
TyG-WC						
Continuous	1.35 (1.08, 1.68)	0.007	1.36 (1.09, 1.69)	0.006	1.33 (1.05, 1.69)	0.017
Q1	Reference		Reference		Reference	
Q2	2.42 (1.11, 5.28)	0.027	2.43 (1.11, 5.31)	0.026	2.41 (1.10, 5.30)	0.029
Q3	3.00 (1.39, 6.43)	0.005	2.99 (1.39, 6.42)	0.005	2.92 (1.34, 6.36)	0.007
Q4	3.19 (1.46, 6.97)	0.004	3.23 (1.48, 7.06)	0.003	3.10 (1.38, 6.96)	0.006
P for trend		0.007		0.006		0.013
TyG-WHtR						
Continuous	1.26 (1.03, 1.54)	0.027	1.26 (1.03, 1.54)	0.025	1.23 (0.99, 1.53)	0.058
Q1	Reference		Reference		Reference	
Q2	1.52 (0.75, 3.06)	0.245	1.51 (0.75, 3.04)	0.252	1.45 (0.71, 2.95)	0.305
Q3	1.91 (0.98, 3.73)	0.058	1.91 (0.98, 3.73)	0.059	1.79 (0.90, 3.56)	0.097
Q4	2.09 (1.08, 4.07)	0.029	2.12 (1.09, 4.12)	0.027	1.98 (0.99, 3.97)	0.053
P for trend		0.027		0.023		0.051
WTI						
Continuous	1.16 (0.95, 1.43)	0.148	1.17 (0.95, 1.43)	0.139	1.15 (0.93, 1.42)	0.205
Q1	Reference		Reference		Reference	
Q2	1.20 (0.63, 2.28)	0.576	1.21 (0.64, 2.30)	0.560	1.20 (0.63, 2.31)	0.580
Q3	1.70 (0.93, 3.09)	0.084	1.70 (0.93, 3.09)	0.083	1.59 (0.86, 2.94)	0.136
Q4	1.45 (0.79, 2.69)	0.233	1.47 (0.79, 2.71)	0.223	1.42 (0.75, 2.68)	0.280
P for trend		0.178		0.172		0.237
LAP						
Continuous	1.14 (0.96, 1.35)	0.140	1.14 (0.96, 1.35)	0.126	1.12 (0.94, 1.34)	0.208
Q1	Reference		Reference		Reference	
Q2	2.04 (1.01, 4.11)	0.046	2.04 (1.01, 4.12)	0.047	2.02 (0.99, 4.09)	0.052
Q3	2.21 (1.12, 4.39)	0.023	2.22 (1.12, 4.41)	0.023	2.15 (1.07, 4.32)	0.033
Q4	2.27 (1.15, 4.50)	0.018	2.30 (1.16, 4.56)	0.017	2.20 (1.09, 4.44)	0.029
P for trend		0.071		0.064		0.114
VAI						
Continuous	1.09 (0.92, 1.29)	0.342	1.09 (0.92, 1.30)	0.310	1.08 (0.90, 1.29)	0.412
Q1	Reference		Reference		Reference	
Q2	1.09 (0.59, 2.03)	0.784	1.09 (0.59, 2.03)	0.785	1.08 (0.57, 2.01)	0.821
Q3	1.35 (0.75, 2.41)	0.314	1.36 (0.76, 2.43)	0.301	1.31 (0.73, 2.36)	0.372
Q4	1.51 (0.86, 2.66)	0.150	1.53 (0.87, 2.69)	0.142	1.44 (0.81, 2.58)	0.216
P for trend		0.114		0.105		0.173
CMI						
Continuous	1.09 (0.91, 1.30)	0.349	1.10 (0.92, 1.31)	0.310	1.07 (0.89, 1.29)	0.443
Q1	Reference		Reference		Reference	
Q2	1.76 (0.93, 3.33)	0.085	1.76 (0.93, 3.35)	0.083	1.83 (0.96, 3.51)	0.067
Q3	1.93 (1.04, 3.59)	0.039	1.94 (1.04, 3.61)	0.037	1.84 (0.97, 3.46)	0.061
Q4	1.55 (0.82, 2.94)	0.177	1.57 (0.83, 2.97)	0.167	1.51 (0.78, 2.93)	0.218
P for trend		0.512		0.492		0.654
AIP						
Continuous	1.14 (0.93, 1.40)	0.196	1.15 (0.94, 1.42)	0.176	1.13 (0.91, 1.40)	0.261
Q1	Reference		Reference		Reference	
Q2	1.16 (0.62, 2.17)	0.633	1.17 (0.63, 2.19)	0.619	1.18 (0.63, 2.23)	0.606
Q3	1.66 (0.93, 2.96)	0.088	1.67 (0.94, 2.98)	0.083	1.64 (0.90, 2.96)	0.104
Q4	1.30 (0.71, 2.39)	0.395	1.31 (0.72, 2.41)	0.379	1.27 (0.68, 2.36)	0.457
P for trend		0.314		0.300		0.391

### 3.3 Multivariable associations of inflammatory and immune–nutritional indices

Among inflammatory and immune–nutritional indices, CTI, the CALLY index, NLR, and PNI had nominal Model 3 associations. CTI was positively associated per 1-SD increment (OR, 1.27; 95% CI, 1.02–1.59; *p* = 0.030). The CALLY index was inversely associated (OR per 1-SD, 0.62; 95% CI, 0.46–0.84; *p* = 0.002); however, the quartile pattern was not uniform because Q3 versus Q1 was not significant (OR, 0.64; *p* = 0.104) and the strongest contrast occurred in Q4 (OR, 0.40; 95% CI, 0.20–0.77; *p* = 0.006). The NLR was positively associated (OR per 1-SD, 1.30; 95% CI, 1.09–1.55; *p* = 0.004), and PNI was inversely associated (OR per 1-SD, 0.74; 95% CI, 0.61–0.91; *p* = 0.004); the PNI Q4 versus Q1 contrast was marginal and did not meet the *p* < 0.05 threshold (OR, 0.57; 95% CI, 0.32–1.01; *p* = 0.054). In the within-family FDR sensitivity analysis, the CALLY index, NLR, and PNI remained significant (all FDR-adjusted *p* = 0.011), whereas CTI did not (FDR-adjusted *p* = 0.068). In the metabolic family, TyG-WC did not remain significant after FDR correction (FDR-adjusted *p* = 0.138). These multiplicity results reinforce the exploratory interpretation of nominal associations (Table 3).

**Table 3:** Multivariable associations of inflammatory and immune–nutritional indices with gallstone disease.

Variable	Model 1		Model 2		Model 3	
	OR (95% CI)	*p*-value	OR (95% CI)	*p*-value	OR (95% CI)	*p*-value
CTI						
Continuous	1.28 (1.04, 1.58)	0.020	1.29 (1.05, 1.59)	0.017	1.27 (1.02, 1.59)	0.030
Q1	Reference		Reference		Reference	
Q2	1.47 (0.72, 2.98)	0.289	1.46 (0.72, 2.96)	0.298	1.45 (0.71, 2.96)	0.307
Q3	2.03 (1.04, 3.98)	0.039	2.01 (1.03, 3.94)	0.042	1.92 (0.97, 3.80)	0.060
Q4	2.19 (1.11, 4.31)	0.023	2.22 (1.13, 4.36)	0.021	2.14 (1.07, 4.30)	0.032
P for trend		0.016		0.014		0.024
RCII						
Continuous	1.14 (0.98, 1.33)	0.084	1.14 (0.98, 1.33)	0.083	1.14 (0.97, 1.33)	0.109
Q1	Reference		Reference		Reference	
Q2	1.12 (0.61, 2.04)	0.720	1.12 (0.61, 2.04)	0.720	1.11 (0.60, 2.03)	0.747
Q3	1.45 (0.81, 2.57)	0.207	1.47 (0.82, 2.61)	0.192	1.39 (0.77, 2.50)	0.268
Q4	1.43 (0.81, 2.50)	0.215	1.45 (0.83, 2.55)	0.195	1.39 (0.78, 2.47)	0.267
P for trend		0.216		0.191		0.272
CALLY index						
Continuous	0.62 (0.46, 0.84)	0.002	0.62 (0.46, 0.83)	0.002	0.62 (0.46, 0.84)	0.002
Q1	Reference		Reference		Reference	
Q2	1.08 (0.68, 1.71)	0.759	1.07 (0.67, 1.70)	0.784	1.04 (0.65, 1.68)	0.863
Q3	0.65 (0.38, 1.12)	0.119	0.64 (0.38, 1.10)	0.104	0.64 (0.37, 1.10)	0.104
Q4	0.40 (0.21, 0.78)	0.007	0.40 (0.21, 0.77)	0.006	0.40 (0.20, 0.77)	0.006
P for trend		0.002		0.002		0.002
NLR						
Continuous	1.29 (1.09, 1.53)	0.004	1.30 (1.09, 1.54)	0.003	1.30 (1.09, 1.55)	0.004
Q1	Reference		Reference		Reference	
Q2	1.47 (0.78, 2.74)	0.232	1.47 (0.78, 2.76)	0.229	1.49 (0.79, 2.81)	0.218
Q3	1.77 (0.96, 3.25)	0.068	1.80 (0.98, 3.31)	0.060	1.79 (0.97, 3.32)	0.064
Q4	2.24 (1.26, 4.01)	0.006	2.27 (1.27, 4.07)	0.006	2.29 (1.27, 4.12)	0.006
P for trend		0.005		0.004		0.004
PLR						
Continuous	1.12 (0.94, 1.35)	0.213	1.12 (0.94, 1.34)	0.213	1.14 (0.95, 1.37)	0.153
Q1	Reference		Reference		Reference	
Q2	0.69 (0.38, 1.24)	0.211	0.69 (0.38, 1.25)	0.218	0.66 (0.36, 1.20)	0.170
Q3	1.12 (0.67, 1.89)	0.663	1.13 (0.67, 1.90)	0.650	1.11 (0.65, 1.87)	0.707
Q4	1.23 (0.74, 2.06)	0.428	1.23 (0.74, 2.07)	0.423	1.26 (0.75, 2.12)	0.388
P for trend		0.194		0.191		0.161
MLR						
Continuous	0.91 (0.75, 1.10)	0.341	0.92 (0.76, 1.12)	0.407	0.92 (0.75, 1.12)	0.394
Q1	Reference		Reference		Reference	
Q2	0.60 (0.34, 1.06)	0.077	0.61 (0.35, 1.06)	0.081	0.62 (0.35, 1.09)	0.099
Q3	0.69 (0.41, 1.17)	0.171	0.70 (0.42, 1.19)	0.190	0.69 (0.41, 1.17)	0.169
Q4	0.75 (0.45, 1.27)	0.286	0.77 (0.46, 1.31)	0.341	0.77 (0.45, 1.32)	0.342
P for trend		0.439		0.505		0.480
SII						
Continuous	1.18 (0.99, 1.41)	0.062	1.19 (1.00, 1.42)	0.053	1.19 (0.99, 1.43)	0.058
Q1	Reference		Reference		Reference	
Q2	0.89 (0.50, 1.60)	0.705	0.91 (0.51, 1.63)	0.747	0.88 (0.49, 1.58)	0.663
Q3	1.17 (0.68, 2.02)	0.559	1.18 (0.69, 2.03)	0.543	1.20 (0.70, 2.08)	0.503
Q4	1.45 (0.86, 2.43)	0.165	1.48 (0.88, 2.50)	0.142	1.39 (0.82, 2.36)	0.220
P for trend		0.090		0.078		0.117
SIRI						
Continuous	1.00 (0.83, 1.20)	0.964	1.01 (0.83, 1.23)	0.909	0.99 (0.81, 1.21)	0.924
Q1	Reference		Reference		Reference	
Q2	0.91 (0.51, 1.63)	0.758	0.92 (0.52, 1.65)	0.785	0.87 (0.48, 1.57)	0.650
Q3	1.30 (0.76, 2.23)	0.333	1.33 (0.78, 2.29)	0.299	1.35 (0.78, 2.33)	0.282
Q4	1.06 (0.61, 1.85)	0.832	1.11 (0.63, 1.95)	0.723	1.02 (0.58, 1.82)	0.939
P for trend		0.620		0.519		0.664
PNI						
Continuous	0.76 (0.62, 0.92)	0.006	0.76 (0.62, 0.93)	0.006	0.74 (0.61, 0.91)	0.004
Q1	Reference		Reference		Reference	
Q2	0.92 (0.57, 1.48)	0.723	0.93 (0.57, 1.50)	0.755	0.93 (0.58, 1.51)	0.780
Q3	0.57 (0.33, 0.99)	0.045	0.57 (0.33, 0.99)	0.047	0.56 (0.33, 0.98)	0.042
Q4	0.58 (0.33, 1.02)	0.061	0.59 (0.33, 1.04)	0.067	0.57 (0.32, 1.01)	0.054
P for trend		0.022		0.024		0.018

### 3.4 Non-linear patterns and single-marker discrimination

**Figure 1:**
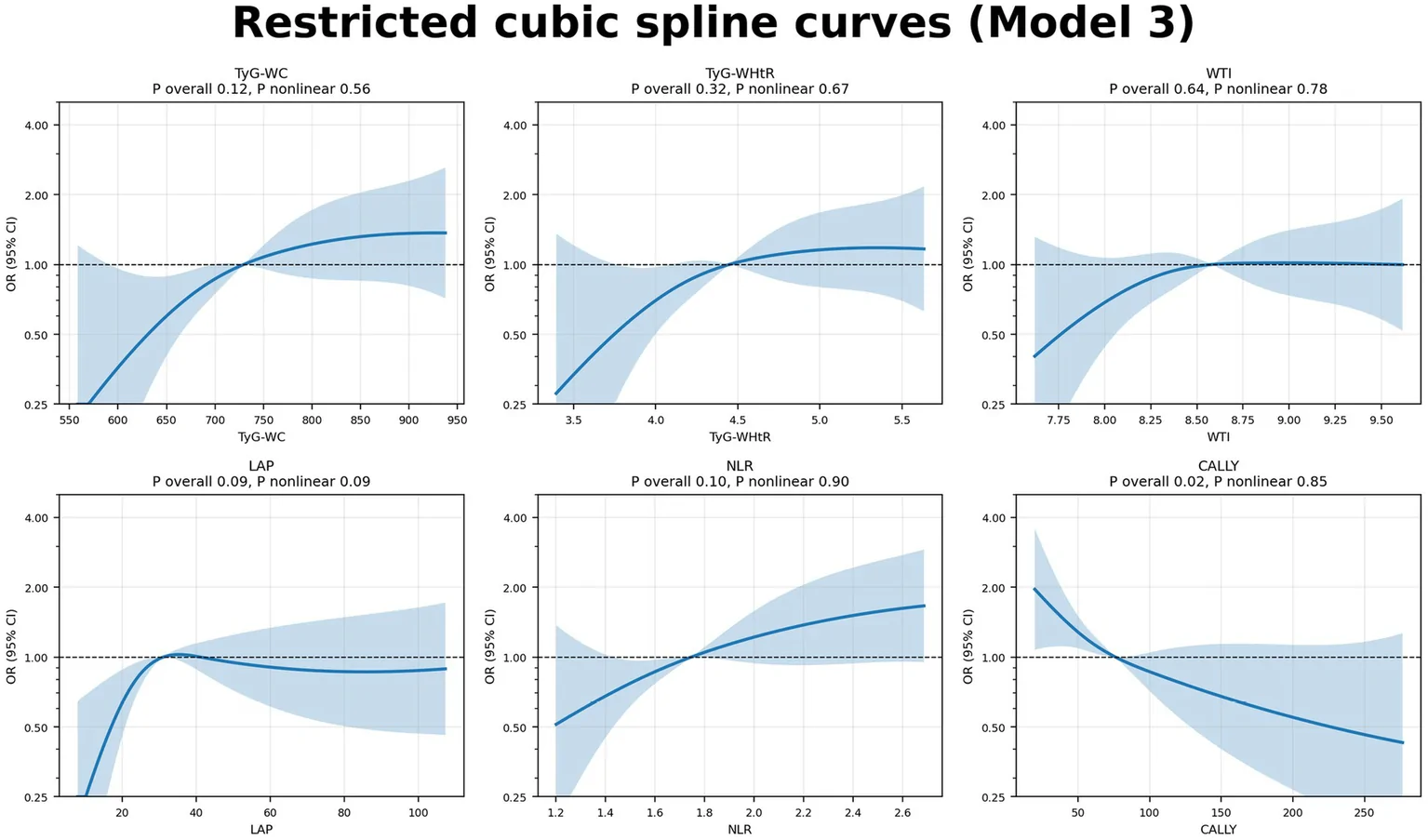
Restricted cubic spline curves for selected indices. Curves were adjusted according to Model 3. P overall and P non-linearity are shown for every panel. Y-axis shows adjusted odds ratio with reference line at 1.0. Curves are displayed across the 2.5th to 97.5th percentiles for visual stability; no visually apparent threshold was interpreted as statistically supported unless the corresponding non-linearity test was significant.

Restricted cubic spline results are reported for all six displayed markers. The CALLY index showed an overall association with gallstone disease (P overall = 0.020) without evidence of non-linearity (P non-linearity = 0.850), consistent with an approximately monotonic inverse pattern over the plotted range. TyG-WC (P overall = 0.120; P non-linearity = 0.560), TyG-WHtR (0.320; 0.670), WTI (0.640; 0.780), LAP (0.090; 0.090), and NLR (0.100; 0.900) did not show statistically significant overall or non-linear spline effects in Model 3. Accordingly, the revised text no longer characterizes visually apparent thresholds as statistically supported non-linear effects (Figure 1).

**Figure 2:**
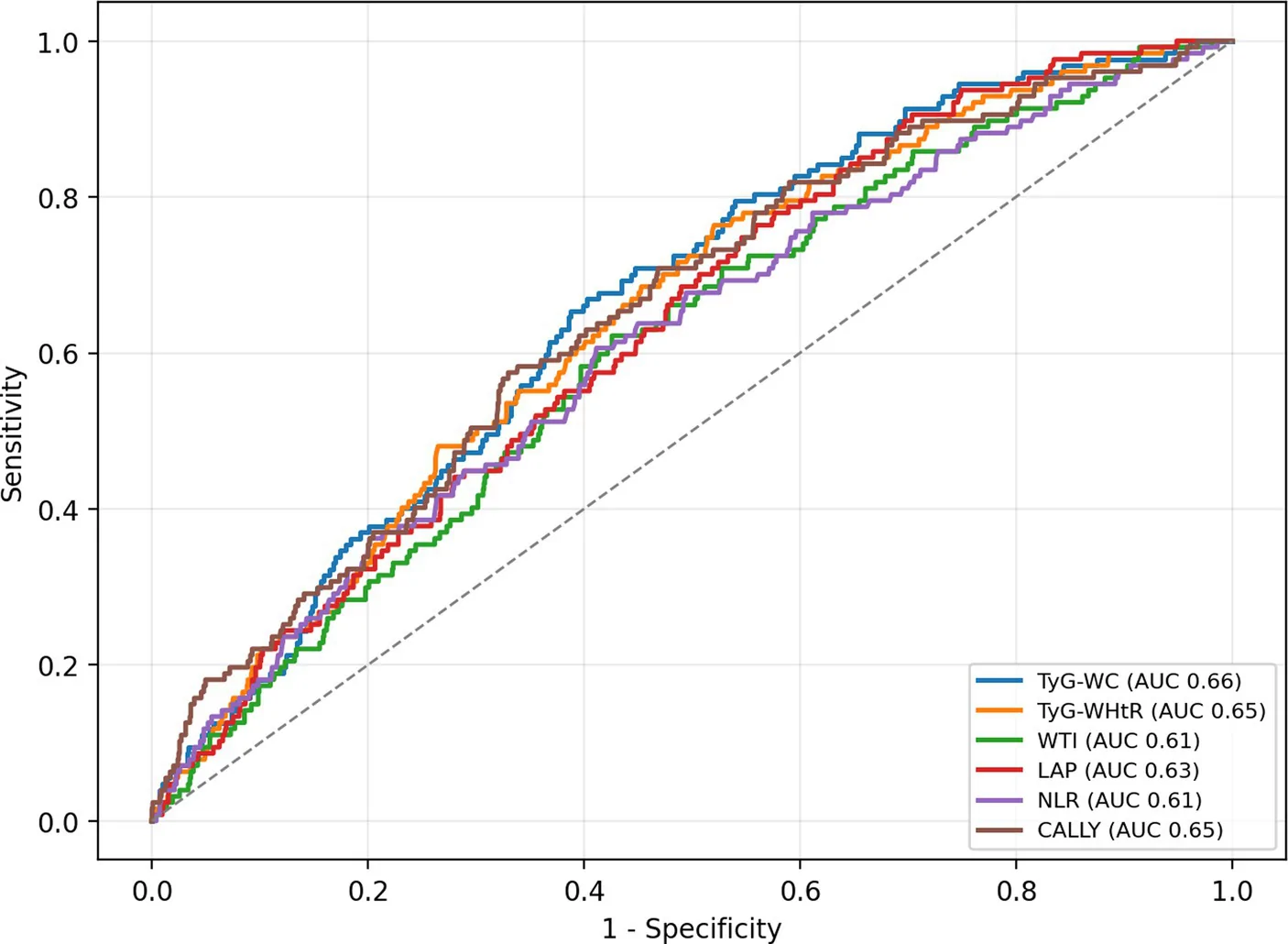
ROC curves for selected individual markers.

**Figure 3:**
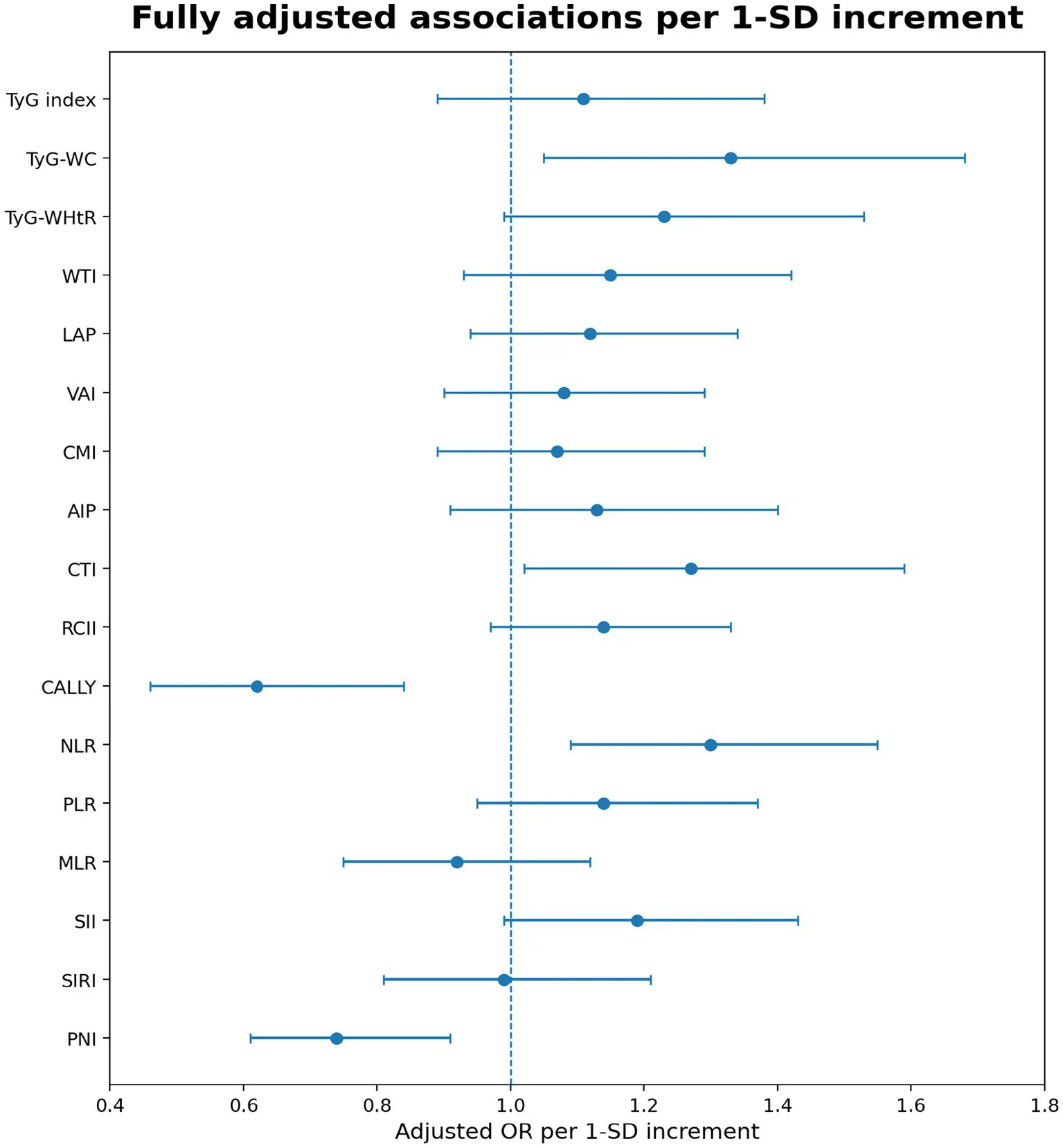
Fully adjusted odds ratios per 1-*SD* increment in candidate indices. Dots indicate adjusted *OR*s, horizontal bars indicate 95% CIs, and the vertical dashed line indicates *OR* = 1.0.

Single-marker ROC analyses showed limited discrimination. TyG-WC had the numerically highest AUC (0.66; 95% CI, 0.61–0.71; *p* < 0.001), followed closely by CTI, the CALLY index, waist circumference, and TyG-WHtR (Table 4 and Figure 2). At the Youden index cutoff for TyG-WC (748.00), sensitivity was 0.67 and specificity 0.60; TyG-WHtR and LAP had higher sensitivity (0.76 for each) but low specificity (0.48 and 0.44, respectively). These operating characteristics are insufficient for stand-alone classification, and the cutoffs should not be interpreted as clinical thresholds. Figure 3 summarizes the fully adjusted associations per 1-SD increment (see Table 5).

**Table 4:** Discrimination of individual indices for gallstone disease.

Index	Direction	AUC (95% CI)	*p*-value	Optimal cutoff	Sensitivity	Specificity
TyG-WC	Higher	0.66 (0.61, 0.71)	<0.001	748.00	0.67	0.60
CTI	Higher	0.65 (0.60, 0.71)	<0.001	9.05	0.54	0.71
CALLY index	Lower	0.65 (0.60, 0.70)	<0.001	58.48	0.57	0.67
Waist circumference	Higher	0.65 (0.60, 0.70)	<0.001	86.90	0.57	0.67
TyG-WHtR	Higher	0.65 (0.59, 0.70)	<0.001	4.38	0.76	0.48
LAP	Higher	0.63 (0.58, 0.69)	<0.001	27.22	0.76	0.44
TyG index	Higher	0.62 (0.56, 0.67)	<0.001	8.62	0.76	0.47
PNI	Lower	0.62 (0.56, 0.67)	<0.001	54.15	0.6	0.61
BMI	Higher	0.62 (0.56, 0.67)	<0.001	24.59	0.63	0.6
NLR	Higher	0.61 (0.56, 0.67)	<0.001	1.82	0.61	0.59
WTI	Higher	0.61 (0.55, 0.66)	<0.001	8.65	0.62	0.57
CMI	Higher	0.61 (0.55, 0.66)	<0.001	0.54	0.70	0.50
VAI	Higher	0.60 (0.55, 0.66)	<0.001	1.74	0.64	0.55
RCII	Higher	0.60 (0.55, 0.66)	<0.001	0.05	0.70	0.46
AIP	Higher	0.59 (0.54, 0.65)	<0.001	0.07	0.62	0.58

**Table 5:** Machine learning model performance in the secondary held-out testing set and stratified 5-fold cross-validation.

Model	Penalized logistic regression	Random forest	Gradient boosting	Extra trees
AUC (95% CI)	0.72 (0.62, 0.81)	0.71 (0.61, 0.80)	0.69 (0.59, 0.79)	0.68 (0.58, 0.78)
*p* value	<0.001	<0.001	<0.001	<0.001
PR-AUC	0.22	0.18	0.18	0.16
Accuracy	0.73	0.81	0.91	0.70
Sensitivity	0.63	0.45	0.03	0.55
Specificity	0.74	0.85	0.99	0.71
Precision	0.20	0.23	0.33	0.16
F1 score	0.30	0.31	0.05	0.25
Brier score	0.19	0.13	0.08	0.19
5-fold cross-validated AUC (95% CI)	0.74 (0.69, 0.78)	0.71 (0.66, 0.76)	0.69 (0.64, 0.73)	0.72 (0.68, 0.77)

### 3.5 Machine learning performance and interpretability

**Figure 4:**
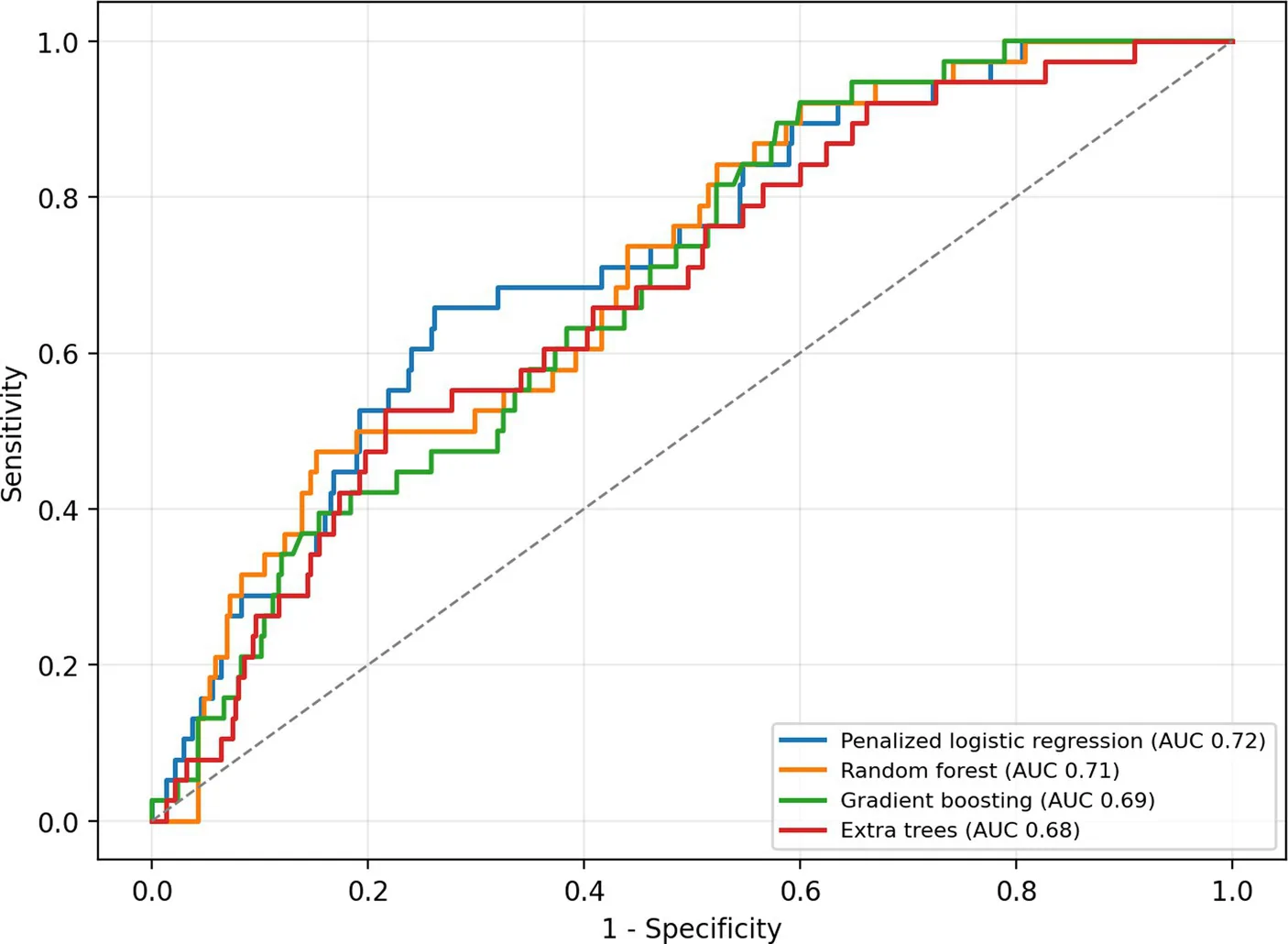
ROC curves for machine learning models in the secondary held-out testing set. These curves are descriptive; stratified 5-fold cross-validation was emphasized for internal validation.

**Figure 5:**
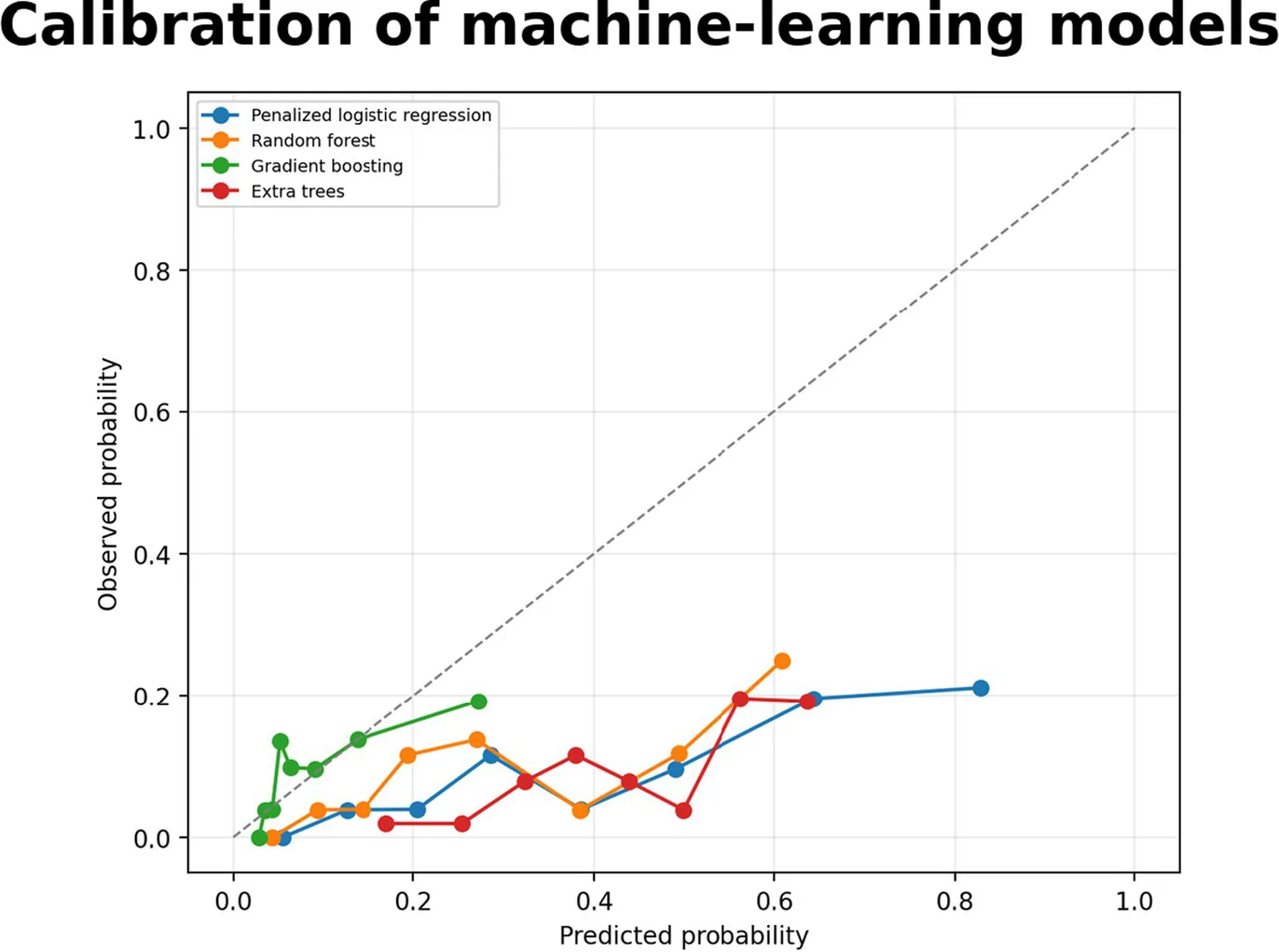
Calibration curves for machine learning models. The diagonal line represents perfect calibration. Several models deviate from this line, especially at higher predicted risks; therefore, the plots do not support use as calibrated clinical prediction tools.

**Figure 6:**
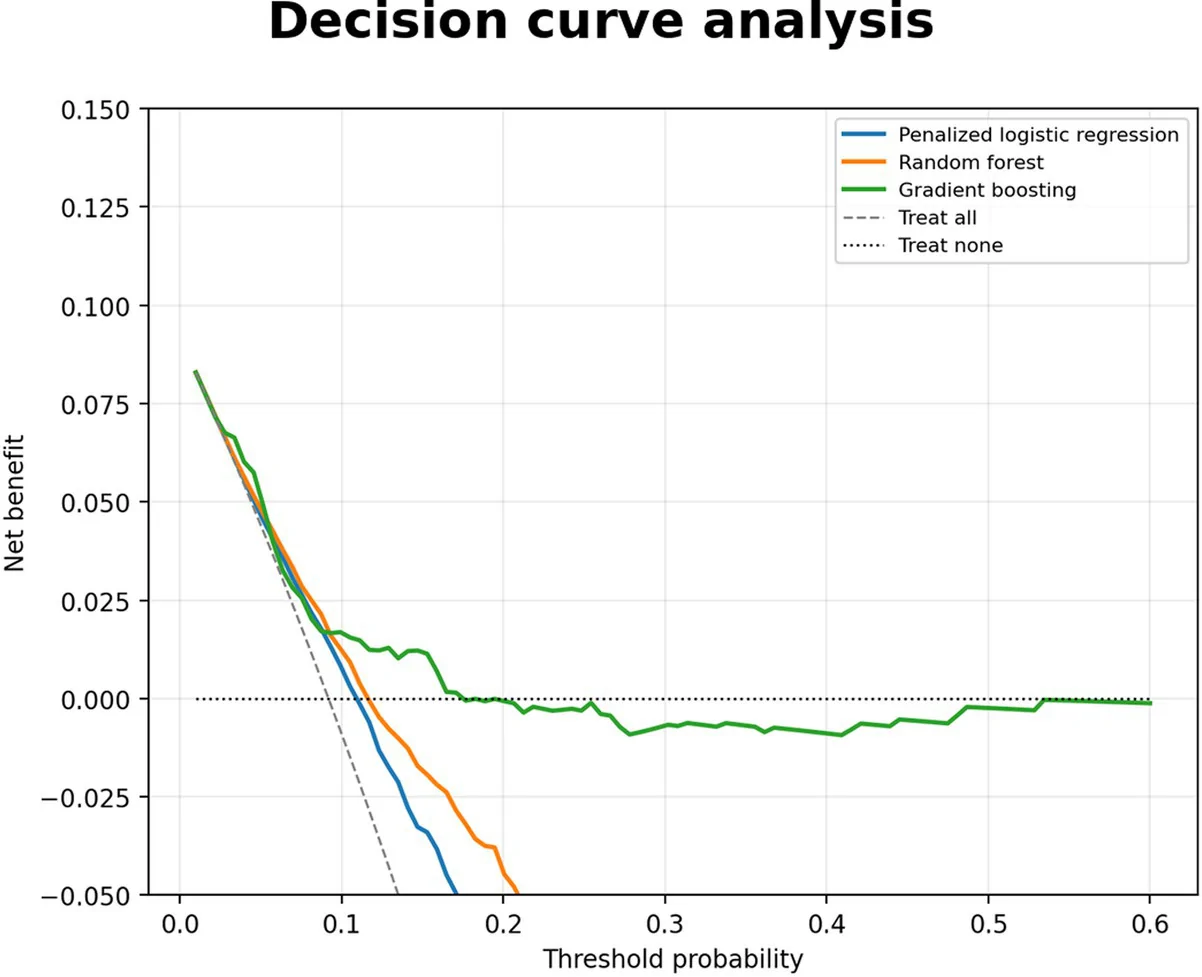
Decision curve analysis for selected prediction models. Net benefit is interpreted relative to treat-all and treat-none strategies and was limited mainly to lower threshold probabilities; the analysis is exploratory because no external validation was performed.

**Figure 7:**
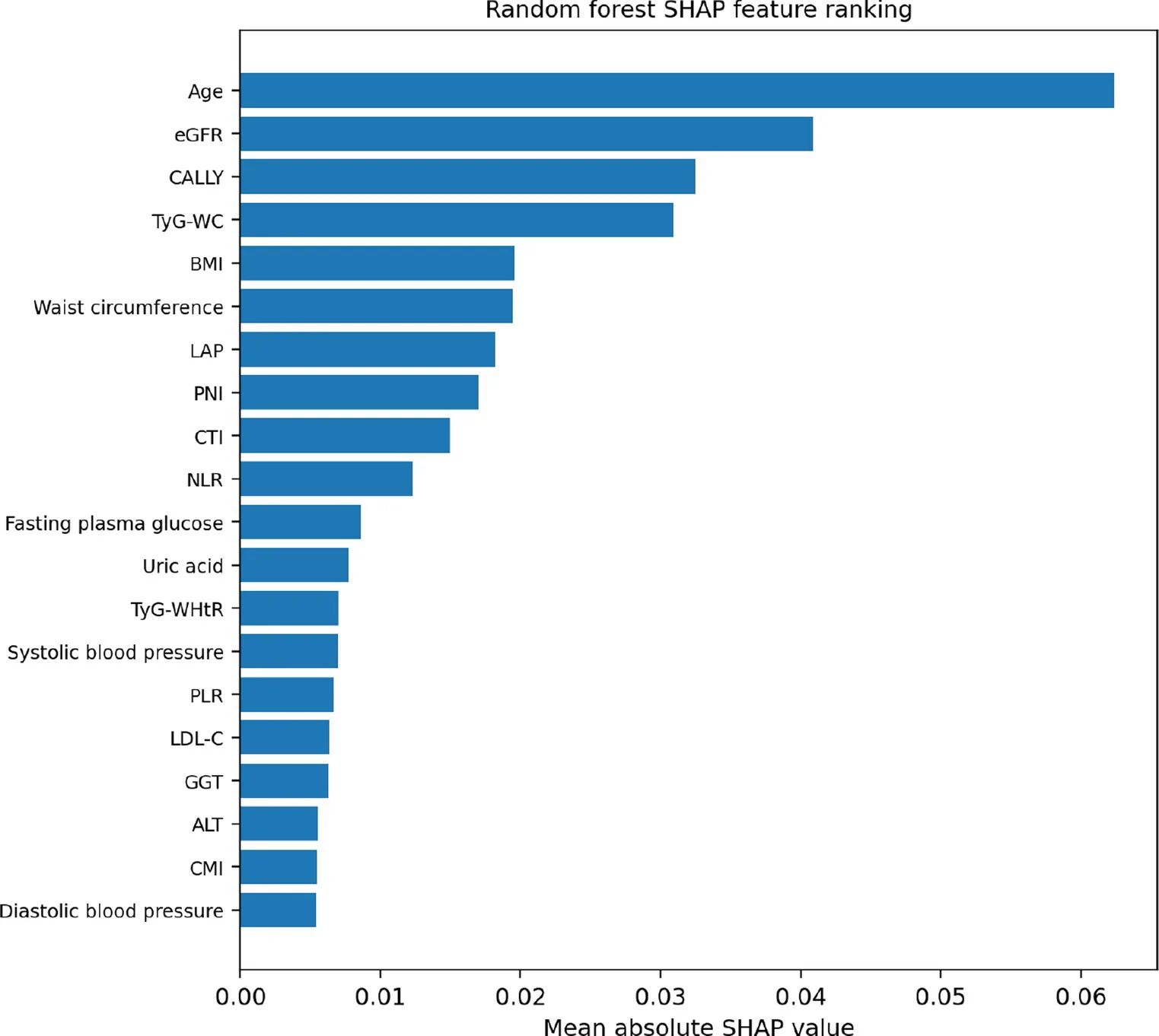
Random forest SHAP feature ranking by mean absolute SHAP value. The ranking represents model-specific contribution magnitude and is not an inferential test.

**Figure 8:**
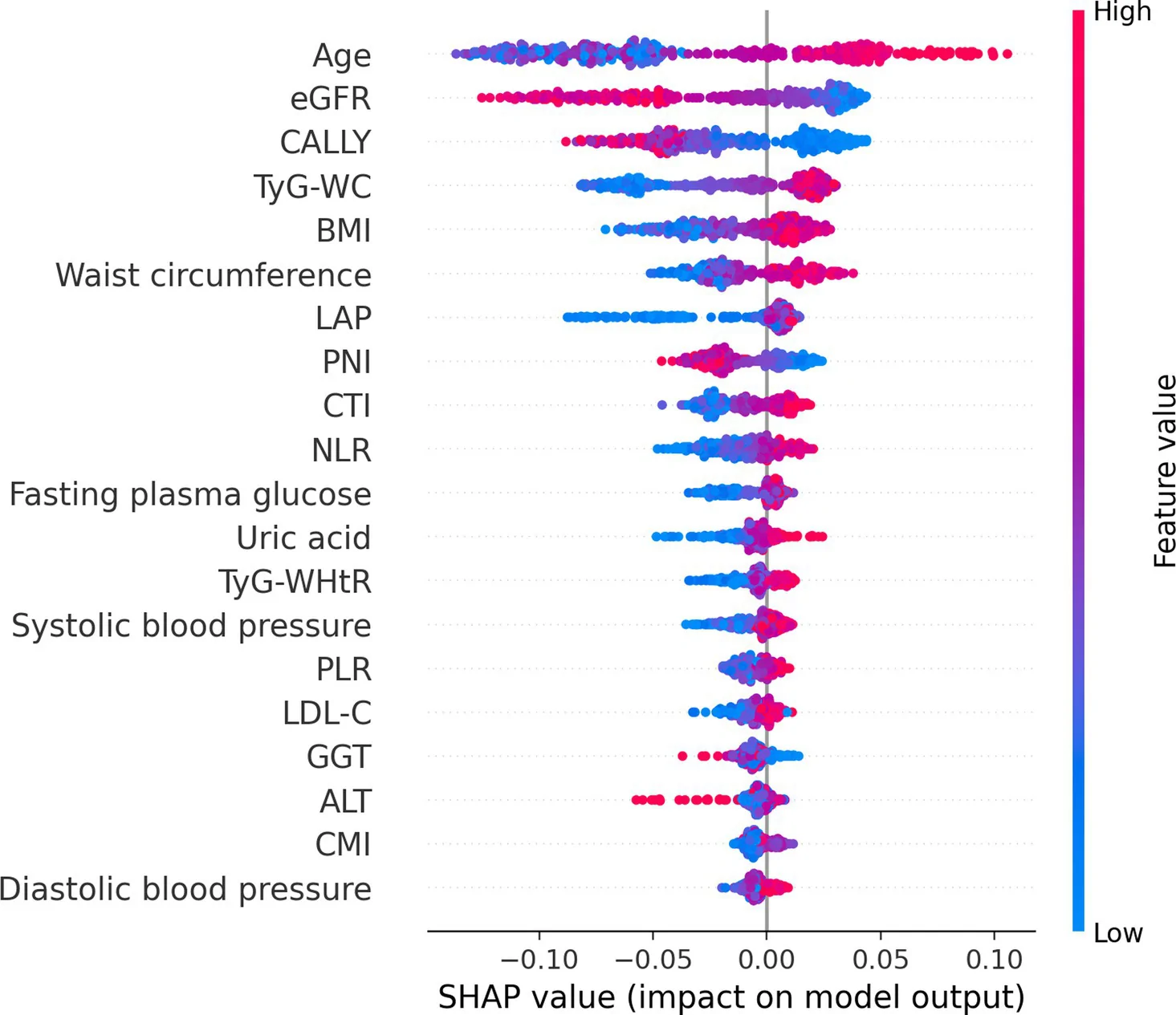
SHAP beeswarm summary plot for the positive gallstone class. For the Scikit-learn random forest classifier, the displayed SHAP values represent contributions to the model probability output for that class; positive values increase and negative values decrease the predicted gallstone probability relative to the model baseline. Feature color represents the observed feature value.

**Figure 9:**
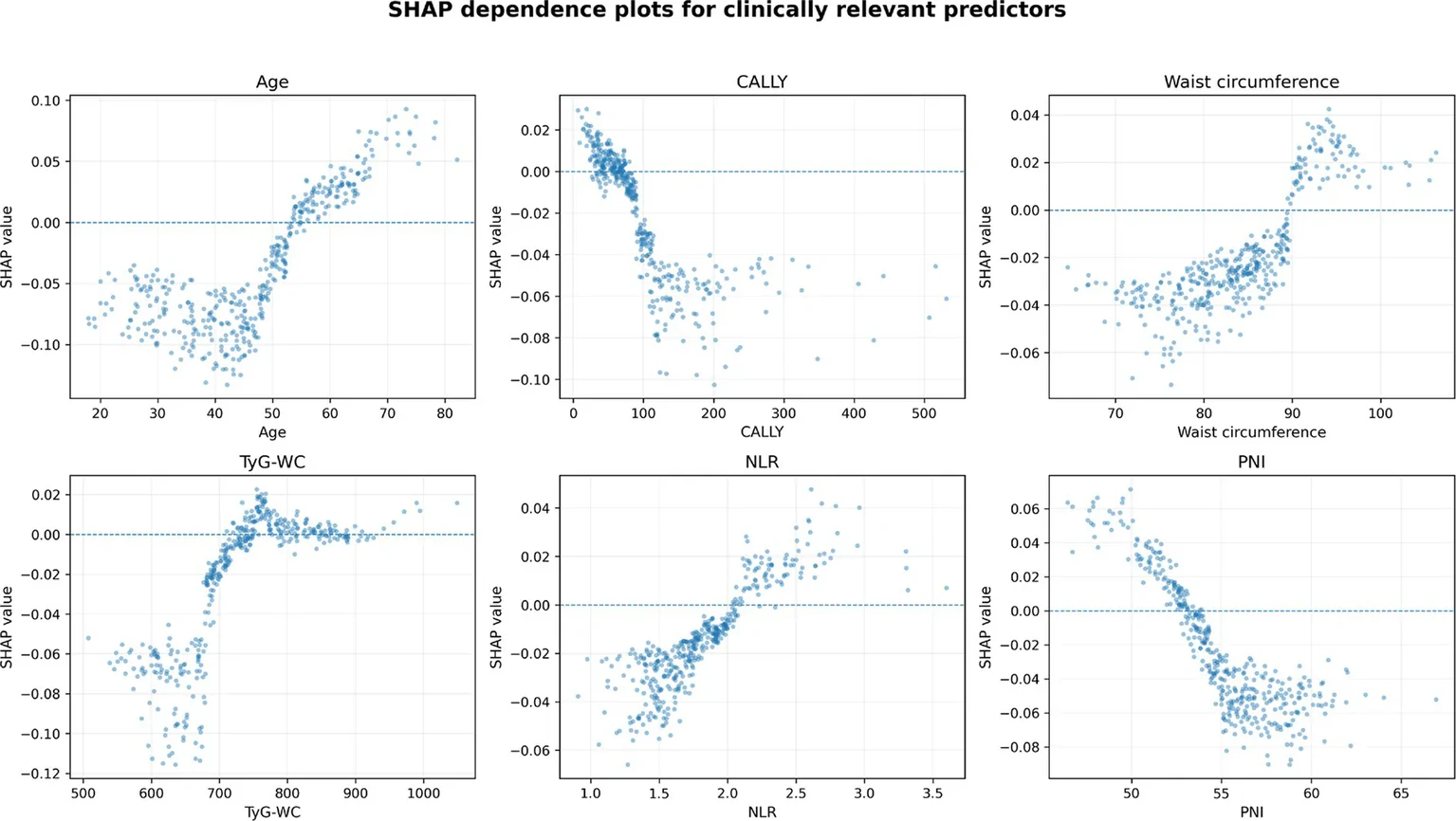
SHAP dependence plots for clinically relevant predictors. Positive SHAP values indicate contributions to a higher model-predicted gallstone probability. Direction is interpreted from the feature–SHAP relationship rather than from the average sign of SHAP values; for the eGFR, lower values tended to contribute to a higher predicted probability, consistent with an inverse dependence pattern. Points are plotted with transparency and slight jitter to reduce overplotting; positive SHAP values indicate higher model-predicted probability.

Machine learning performance was modest and should be interpreted in the context of substantial class imbalance. In the secondary held-out testing set, penalized logistic regression had an AUC of 0.72 (95% CI, 0.62–0.81), with a sensitivity of 0.63, specificity of 0.74, precision of 0.20, and F1 score of 0.30. Thus, approximately four of five positive classifications at the reported threshold were false positives. Gradient boosting achieved an apparently high accuracy of 0.91 but sensitivity of only 0.03, indicating that accuracy was dominated by the prediction of the majority non-gallstone class. Its low Brier score (0.08) should therefore not be read in isolation as evidence of useful case detection. PR-AUC values ranged from 0.16 to 0.22, compared to an outcome prevalence of 0.092, indicating improvement over the prevalence-level baseline but still limited positive-class discrimination. In stratified 5-fold cross-validation, AUC values remained modest: Penalized logistic regression, 0.74 (95% CI, 0.69–0.78); random forest, 0.71 (0.66–0.76); gradient boosting, 0.69 (0.64–0.73); and extra trees, 0.72 (0.68–0.77). Calibration curves showed deviations from ideal calibration, particularly at higher predicted probabilities, and decision curve net benefit was limited mainly to lower thresholds (Figures 4–6). SHAP analysis ranked age, eGFR, the CALLY index, TyG-WC, BMI, and waist circumference among the leading contributors to random forest output (Table 6; Figures 7–9). SHAP directions were interpreted from dependence plots rather than from mean signed SHAP values. For example, lower eGFR tended to contribute to higher model output, representing an inverse feature–prediction pattern rather than a negative SHAP contribution per se.

**Table 6:** Random forest SHAP-ranked predictors and qualitative dependence patterns for gallstone model output.

Feature	Mean absolute SHAP value	Dependence pattern	Interpretation
Age	0.06	Higher age tended to increase model output	Model-specific, not causal
eGFR	0.04	Lower eGFR tended to increase model output	Inverse dependence; requires external replication
CALLY index	0.03	Lower CALLY index values tended to increase model output	Consistent with an inverse regression association
TyG-WC	0.03	Higher TyG-WC tended to increase model output	Consistent with a positive regression association
BMI	0.02	Higher BMI tended to increase model output	Correlated with central adiposity measures
Waist circumference	0.02	Higher waist tended to increase model output	Correlated with TyG-WC and LAP
LAP	0.02	Higher values generally increased model output	Lower-ranked dependence; interpret cautiously
PNI	0.02	Lower values generally increased model output	Inverse dependence
CTI	0.01	Higher values generally increased model output	Nominal regression association; FDR not significant
NLR	0.01	Higher values generally increased model output	Consistent with a positive regression association
Fasting plasma glucose	0.01	Mixed or weak dependence at the observed range	No directional inference emphasized
Uric acid	0.01	Mixed or weak dependence at the observed range	No directional inference emphasized
TyG-WHtR	0.01	Mixed or weak dependence at the observed range	No directional inference emphasized
Systolic blood pressure	0.01	Mixed or weak dependence at the observed range	No directional inference emphasized
PLR	0.01	Mixed or weak dependence at the observed range	No directional inference emphasized
LDL-C	0.01	Mixed or weak dependence at the observed range	No directional inference emphasized
GGT	0.01	Mixed or weak dependence at the observed range	No directional inference emphasized
ALT	0.01	Mixed or weak dependence at the observed range	No directional inference emphasized
CMI	0.01	Mixed or weak dependence at the observed range	No directional inference emphasized
Diastolic blood pressure	0.01	Mixed or weak dependence at the observed range	No directional inference emphasized

Mean absolute SHAP values quantify model-specific contribution magnitude. Mean signed SHAP values and univariable p-values are omitted because neither should be used as a substitute for association direction or inferential significance. Dependence pattern descriptions are qualitative model explanations and are not causal effects.

## 4 Discussion

In this single-center, cross-sectional health examination cohort, 9.23% of participants had ultrasonography-detected gallstones. The principal findings were that TyG-WC showed a nominal association with gallstone disease after multivariable adjustment, while the CALLY index, NLR, and PNI showed associations that remained significant in the within-family FDR sensitivity analysis. The metabolic findings were sensitive to adjustment for age and to multiplicity correction, and several quartile estimates were imprecise. We therefore interpret the results as evidence of potentially relevant metabolic and immune–nutritional correlates rather than as proof that any composite index is superior to conventional risk factors.

The TyG-WC association is biologically plausible because the index combines triglyceride–glucose burden with central adiposity, two features linked to insulin resistance and altered hepatic lipid handling (7–10, 17–20). However, biological plausibility does not resolve confounding or establish temporality in a cross-sectional study. The marked attenuation from the crude TyG-WC estimate to the age- and sex-adjusted estimate illustrates the importance of demographic confounding in this dataset, and the FDR result further argues against describing TyG-WC as a definitive or uniquely robust marker.

The findings are compatible with a metabolic-inflammatory framework in which insulin resistance, central adiposity, inflammatory activity, and nutritional–inflammatory status may coexist in individuals with gallstones. These pathways could influence hepatic cholesterol handling, bile composition, gallbladder motility, or mucosal inflammatory responses, but the present data do not distinguish confounding from mediation and do not measure these mechanisms directly. We therefore use mechanistic discussion only to contextualize the observed associations rather than to infer a causal sequence.

**Figure 10:**
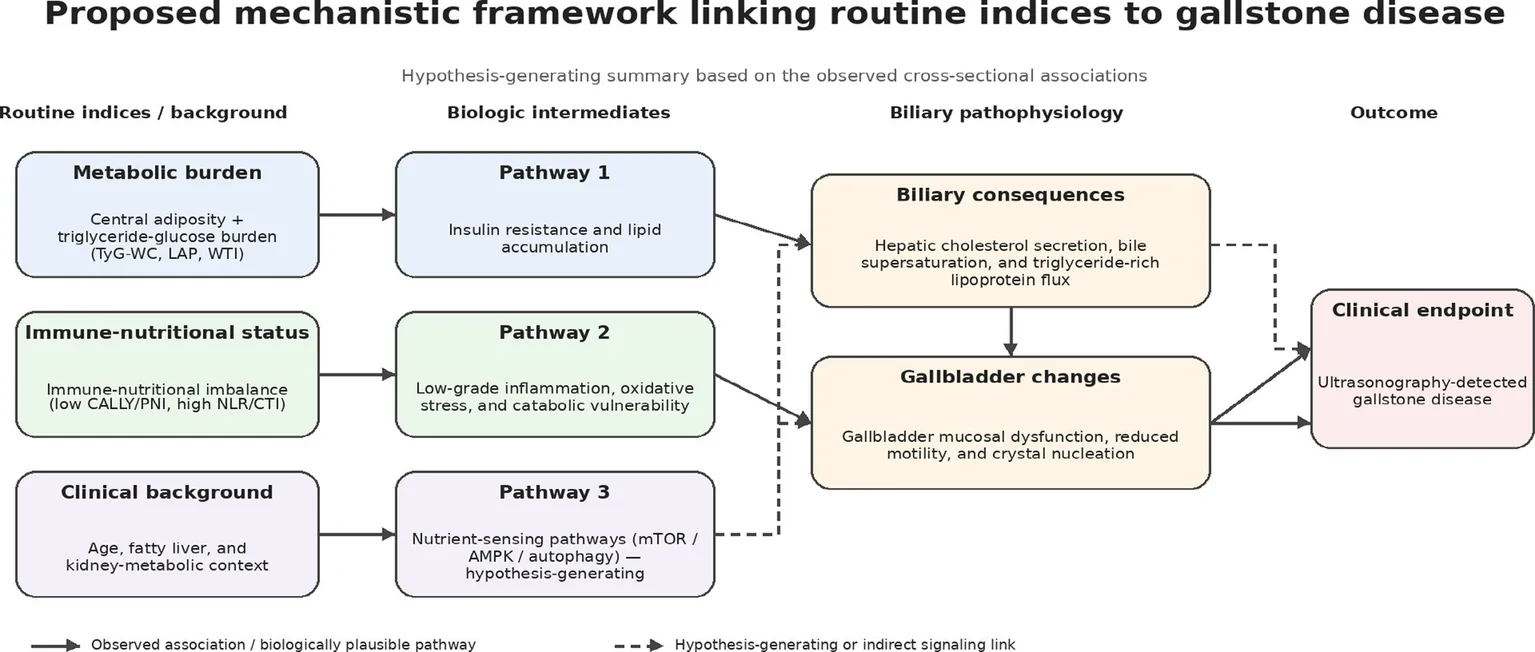
Hypothesis-generating conceptual framework linking metabolic burden, immune–nutritional status, nutrient-sensing pathways, inflammation, and ultrasonography-detected gallstone disease. None of the intermediate biological pathways in this schematic were directly measured in the present cohort. TyG-WC, triglyceride-glucose waist circumference index; LAP, lipid accumulation product; WTI, waist triglyceride index; CALLY, C-reactive protein-albumin-lymphocyte index; PNI, prognostic nutritional index; NLR, neutrophil-to-lymphocyte ratio; CTL, C-reactive protein-TyG index. mTOR signaling was not directly measured in this cohort, the signaling component is intended only as a mechanistic interpretation ald.

Nutrient-sensing pathways, such as mTOR, provide one possible bridge between metabolic state and inflammatory signaling through effects on hepatic lipid metabolism, glucose handling, autophagy, and cellular stress responses (31–37). As mTOR activity, bile composition, gallbladder contractility, and tissue-level inflammatory pathways were not measured, this mechanism remains hypothesis-generating. The revised discussion removes tangential signaling examples that were not directly relevant to gallstone disease and limits Figure 10 to a conceptual framework rather than a mechanistic claim.

Our findings should be positioned alongside, rather than above, recent population-based studies. Several studies cited here used NHANES 2017–2020 and defined gallstone history based on questionnaire data (11–16), whereas other studies in the gallstone literature included imaging-based cohorts. The value of the present dataset is, therefore, its independent single-center setting, concurrent ultrasonography-based outcome ascertainment, and simultaneous evaluation of metabolic and immune–nutritional indices. We have removed the earlier overgeneralized statement that prior studies broadly lacked imaging-based definitions.

The study compares multiple routinely derived indices within the same cohort, but it did not formally test whether any index provided predictive information beyond a conventional clinical model containing age, sex, BMI, glucose, and lipids. Consequently, terms such as “most informative,” “superior,” and “incremental predictive value” are not supported by the present analyses and have been removed. The ROC and machine learning analyses should instead be viewed as descriptive comparisons of discrimination and model contribution within this dataset.

WTI illustrates the importance of adjustment. Although the crude WTI association was positive, it was no longer statistically significant after age and sex adjustment and remained non-significant in Model 3. We therefore do not attribute its attenuation to any single covariate or imply whether the adjusted variables are confounders, mediators, or collinear correlates. Similarly, the discrepancy between continuous and quartile LAP results may reflect categorization, sampling variability, or distributional features; the spline analysis did not provide statistically persuasive evidence for a non-linear LAP effect.

The CALLY index, NLR, and PNI showed the most consistent immune–nutritional associations after FDR correction, but their effect sizes were moderate and do not, by themselves, establish clinical usefulness. The CALLY quartile pattern was strongest in Q4 and did not show significance across all intermediate quartiles, while the PNI Q4 contrast was marginal. These results support further investigation of systemic inflammatory and nutritional status in gallstone populations, but they should not be interpreted as establishing clinically actionable thresholds.

The prediction analyses do not support clinical deployment. A single-marker AUC of 0.66 indicates limited discrimination, and the machine learning AUC values remained below 0.75 during internal validation. Low PR-AUC values, low positive predictive value, class imbalance effects, imperfect calibration, and restricted decision curve net benefit further limit clinical interpretation. The eGFR SHAP ranking should also be treated cautiously. The eGFR was included as an exploratory model predictor and an extended adjustment variable, rather than as a prespecified mechanistic gallstone factor. Its importance may reflect age, comorbidity structure, correlated predictors, or model-specific partitioning and requires independent replication before biological interpretation.

Strengths of the study include objective ultrasonography-based outcome ascertainment, measured anthropometry, a complete final analytic dataset, and evaluation of several metabolic and immune–nutritional indices derived from routine laboratory data. We also report multivariable effect estimates, quartile analyses, spline tests, ROC operating characteristics, internal cross-validation, calibration, decision curves, and SHAP explanations. These complementary analyses are useful for triangulation, but they do not convert an exploratory cross-sectional study into a validated prediction study.

Several important limitations must be acknowledged. First, the cross-sectional design precludes temporal and causal inference and allows for reverse causation. Second, the consecutive convenience sample was drawn from a single health examination center in China. Exclusions due to unavailable ultrasonography, missing variables, and other data-quality reasons may have introduced selection bias, and the external generalizability of the findings remains uncertain. Third, medication history, long-term dietary patterns, parity, estrogen exposure or hormone replacement therapy, detailed reproductive history, disease duration, gallstone composition, and other potentially relevant factors were unavailable. In women, the absence of parity and estrogen-related variables is particularly important because these factors may be associated with gallstone risk; their omission may therefore produce residual confounding and should not be assumed to be negligible. Fourth, the published LAP constants were derived from populations outside the present study population and were not recalibrated for this Chinese cohort. Fifth, many related indices and secondary analyses were examined; although within-family FDR sensitivity analyses were performed, multiplicity and model selection uncertainty remain. Finally, the machine learning results were internally evaluated only, were affected by the low event prevalence and class imbalance, and should not be used for clinical decisions without external validation, prospective assessment, and formal evaluation of incremental value beyond conventional predictors.

## 5 Conclusion

Among adults undergoing routine health examinations, several metabolic and immune–nutritional indices were associated with prevalent ultrasonography-detected gallstone disease. TyG-WC showed a nominal multivariable association, whereas the CALLY index, NLR, and PNI remained associated after within-family FDR correction. Single-marker and machine learning discrimination was modest, and the study did not assess incremental value beyond conventional clinical predictors. The findings should therefore be regarded as hypothesis-generating and require multicenter external validation, longitudinal assessment, and formal evaluation of clinical utility before they can be considered for use in risk stratification.

## Data Availability

The original contributions presented in the study are included in the article/Supplementary material, further inquiries can be directed to the corresponding author.
