# Supplementary material for: Cross-sectional associations of lipid accumulation and immune–nutritional indices with ultrasonography-detected gallstone disease: an explainable machine learning analysis
Source: Front Nutr. 2026 Sep 9;13:1879120. doi: 10.3389/fnut.2026.1879120 (PMC13597763; doi:10.3389/fnut.2026.1879120)
Supplement: Supplementary file 1 [file Table_1.docx]

**Supplementary Table S1. Definitions and formulas of derived indices**

| **Index** | | **Formula** | **Unit / note** |
| --- | --- | --- | --- |
| **BMI** | Weight (kg) / height (m)^2 | | Anthropometric |
| **WHtR** | Waist circumference / height | | Same length units |
| **WTI** | ln[TG (mg/dL) x waist circumference (cm) / 2] | | TG converted to mg/dL |
| **TyG** | ln[TG (mg/dL) x fasting glucose (mg/dL) / 2] | | Metabolic surrogate |
| **TyG-BMI** | TyG x BMI | | Composite |
| **TyG-WC** | TyG x waist circumference (cm) | | Composite |
| **TyG-WHtR** | TyG x WHtR | | Composite |
| **LAP, men** | [waist circumference (cm) - 65] x TG (mmol/L) | | Original published constant |
| **LAP, women** | [waist circumference (cm) - 58] x TG (mmol/L) | | Original published constant |
| **VAI, men** | [WC/(39.68 + 1.88 x BMI)] x (TG/1.03) x (1.31/HDL-C) | | TG and HDL-C in mmol/L |
| **VAI, women** | [WC/(36.58 + 1.89 x BMI)] x (TG/0.81) x (1.52/HDL-C) | | TG and HDL-C in mmol/L |
| **CMI** | (TG/HDL-C) x WHtR | | TG and HDL-C in mmol/L |
| **AIP** | log10(TG/HDL-C) | | TG and HDL-C in mmol/L |
| **RC** | Total cholesterol - LDL-C - HDL-C | | mmol/L |
| **RCII** | RC x hsCRP / 10 | | Composite |
| **CTI** | TyG + 0.412 x ln(hsCRP) | | hsCRP in mg/L |
| **CALLY** | Albumin x lymphocyte count / hsCRP | | Albumin g/L; lymphocytes 10^9/L; hsCRP mg/L |
| **NLR** | Neutrophil count / lymphocyte count | | Cell-count ratio |
| **PLR** | Platelet count / lymphocyte count | | Cell-count ratio |
| **MLR** | Monocyte count / lymphocyte count | | Cell-count ratio |
| **SII** | Platelet x neutrophil / lymphocyte | | Cell-count composite |
| **SIRI** | Neutrophil x monocyte / lymphocyte | | Cell-count composite |
| **PNI** | Albumin (g/L) + 5 x lymphocyte count (10^9/L) | | Immune-nutritional index |
